# The Use of Model Cellulose Materials for Studying Molecular Interactions at Cellulose Interfaces

**DOI:** 10.1021/acsmacrolett.3c00578

**Published:** 2023-11-01

**Authors:** Nadia Asta, Michael S. Reid, Torbjörn Pettersson, Lars Wågberg

**Affiliations:** † Department of Fibre and Polymer Technology, KTH Royal Institute of Technology, Teknikringen 58, SE-100 44 Stockholm, Sweden; ‡ RISE Research Institute of Sweden, SE-114 86 Stockholm, Sweden; § Wallenberg Wood Science Centre, Department of Fibre and Polymer Technology, KTH Royal Institute of Technology, Teknikringen 56, 10044 Stockholm, Sweden

## Abstract

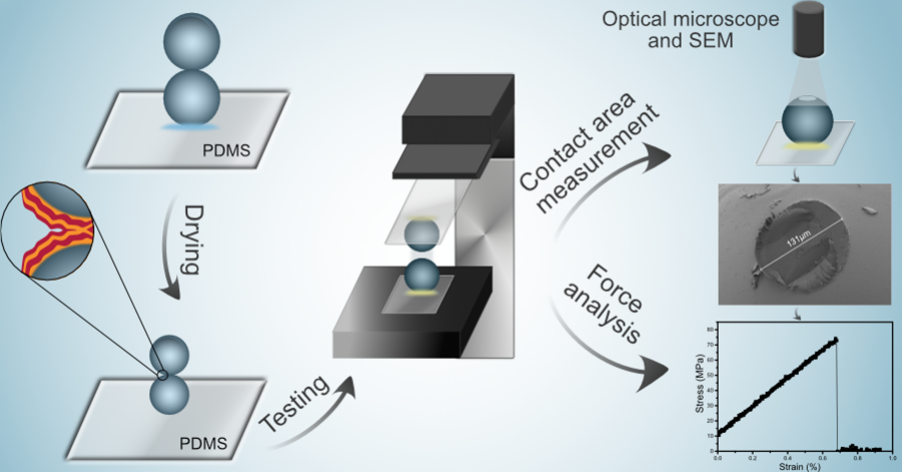

Despite extensive research on biobased and fiber-based materials, fundamental questions regarding the molecular processes governing fiber–fiber interactions remain unanswered. In this study, we introduce a method to examine and clarify molecular interactions within fiber–fiber joints using precisely characterized model materials, i.e., regenerated cellulose gel beads with nanometer-smooth surfaces. By physically modifying these materials and drying them together to create model joints, we can investigate the mechanisms responsible for joining cellulose surfaces and how this affects adhesion in both dry and wet states through precise separation measurements. The findings reveal a subtle balance in the joint formation, influencing the development of nanometer-sized structures at the contact zone and likely inducing built-in stresses in the interphase. This research illustrates how model materials can be tailored to control interactions between cellulose-rich surfaces, laying the groundwork for future high-resolution studies aimed at creating stiff, ductile, and/or tough joints between cellulose surfaces and to allow for the design of high-performance biobased materials.

Cellulose-rich fibrous networks are ubiquitous in everyday life, both macroscopically in different packaging and hygiene products, but also in biocomposites and nonwoven materials that are used in a variety of products from simple wipes to medical dressing gowns.^1^ During the last two decades, there has also been a rapid development of materials made from nanocellulose,^2^ where the high anisotropy, ease of chemical modification, and excellent mechanical properties of these materials have spurred a virtual avalanche of research and development. Nanocelluloses have been applied to create aerogels, nanopapers, energy storage devices, and responsive membranes just to mention a few.^2^ Within many of these applications, the cellulose/cellulose interactions are imperative in order to utilize the inherent properties of the nanocelluloses.

However, despite the obvious need for an understanding of the molecular interactions between cellulose-rich surfaces, there is today no fundamental understanding of the mechanism controlling these interactions.^3^ One obvious misconception is that hydrogen bonding is responsible for strong interactions at all length scales due to the abundance of hydroxyl groups. Yet, considering the short-range and specificity of these interactions, this is a too simple and too blunt description.^4^ One major reason for the lack of fundamental data is the chemical and physical heterogeneity of cellulose-rich fibers, making them rather challenging for the determination of molecular interactions. However, the development of smooth and chemically well-characterized model cellulose surfaces^5−7^ has paved the way for more fundamental studies of cellulose/cellulose interactions. It was previously found^8−10^ that dispersive van der Waals forces greatly influence interactions between surfaces (cellulose, hemicellulose, and lignin) under dry conditions, but in wet and moist conditions, swelling and capillary condensation begin to dominate. The large influence of water content during the preparation of cellulose-rich materials on final materials properties has also been shown in a recent review.^11^

These investigations show that the concept of explaining the interactions between cellulose-rich surfaces by hydrogen bonding is an oversimplification and that van der Waals interactions have a large influence on the interactions. Moreover, the interactions between wet cellulose surfaces are also greatly affected by the swelling of the surface. It is also essential to determine the influence of how the water is removed during drying and how the mechanical properties of the outer layers of the surfaces are affected by water removal. This later finding was also supported by rather recent investigations regarding the effect of strength-enhancing additives on cellulose and PDMS model surfaces.^12^ These investigations showed that there is a large influence of the additives on the wet/drying properties of the cellulose surfaces.^13,14^ The inherent properties of the additives (wet/dry) will significantly influence the interfacial structure and hence the molecular contact zone between the surfaces and naturally affect the overall final adhesion. These principal results are also in accordance with earlier studies of the fundamental interactions between polybutylmethacrylate surfaces that were joined together under different temperatures,^15^ indicating how the complex interphase would develop between cellulose surfaces when water is removed during drying.

Taking this all together means that in order to study the molecular interactions between two cellulose-rich surfaces during drying, it is necessary to have access to initially smooth model cellulose surfaces with controlled physical and chemical properties. During the last years there has fortunately been an interesting development of macroscopic cellulose surfaces with a spherical shape and a nanometer smooth surface allowing for these types of studies.^16,17^ By using this model system, we have been able to study the fundamental interactions between cellulose-rich surfaces during drying and how the separation force after drying will be affected by treatments and testing conditions. These materials can then, under well-controlled conditions, be modified and characterized, dried together, and tested for adhesion, and finally, the contact zone between the materials can be analyzed both before and after separation.

Despite the wide use of strength additives in fiber-based materials, it is unclear how, for example, a LbL assembly of polyelectrolytes on the surface of the fibers impacts the molecular interactions of cellulose–cellulose joints. Moreover, to date, there has yet to be a demonstration of a model experiment that can accurately provide molecular-scale insight into cellulose–cellulose interactions. In this study, a model system using smooth macroscopic cellulose beads is presented. The force required to separate the model joints with and without a well-defined LbL assembly of poly(allylamine hydrochloride) (PAH) and hyaluronic acid (HA) is evaluated. The separation stress is then determined by normalizing the force by the contact area determined after separation.

Cellulose beads were prepared by dripping a cellulose-LiCl/DMAc (lithium chloride dimethylacetamide) solution into a regeneration bath of ethanol (see Experimental section in SI).^16,18^ The resulting nanometer-smooth cellulose beads, Figure 1, were dried and reswollen prior to joint formation. Cellulose beads were prepared from cellulose-rich fibers with different degrees of oxidation. The oxidation creates carboxylic acid groups attached to the cellulose, but at degrees of substitution significantly below the level needed to dissolve the cellulose in water. The wet diameters of the never-dried cellulose beads are 1505 ± 24 μm for the low charged (29 μeq/g), 1590 ± 32 μm for the medium charged (300 μeq/g), and 1679 ± 65 μm for the high charged (600 μeq/g) beads. When dried, the diameter of the beads shrinks to approximately 500 μm and when reswollen in 10 mM NaCl, reswells to 53%, 55%, and 61%, respectively, of the original size, going from lowest to highest charge density.

**Figure 1:**
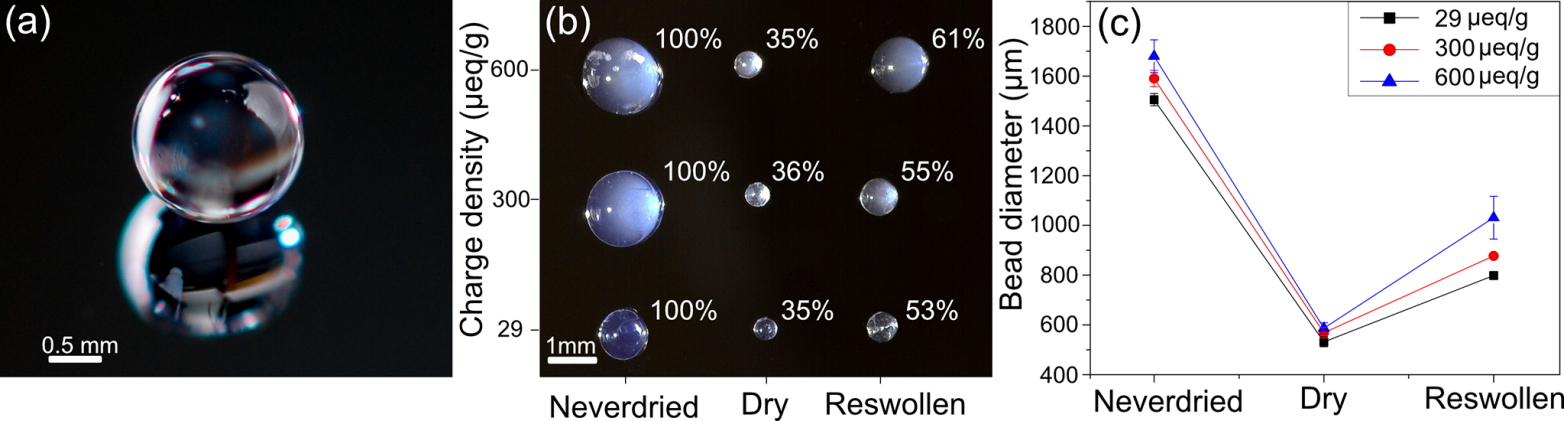
(a) Side-view of a wet bead to clarify that the apparent opacity of the beads as seen in (b) is a macroscopic optic effect and not from an internal or surface structure of the beads. (b) Regenerated cellulose beads with a charge density (carboxylic acid groups) of the cellulose of 29, 300, and 600 μeq/g, before drying and after drying and reswelling of the beads in water with Na^+^ as counterions to the charges and the corresponding sizes of the beads (c).

The permanent change in swelling of the beads following the drying step has also been demonstrated earlier and is referred to as a hornification of the cellulose.^18,19^ However, as shown before, the dry beads are very smooth, with a surface roughness in the nm-range.^16^ Moreover, when dried from water and reswollen in water or low ionic strength aqueous media, this smoothness is preserved. This makes them ideal for adhesion studies, where the surface roughness is a very important factor.

To understand molecular interactions, it is essential to determine how the charge of the beads will influence the wet modulus and if and how additives affect these properties. In previous work^18^ the elasticity of the wet beads was evaluated with AFM (atomic force microscopy) indentation techniques (using a 10 μm spherical probe (diameter) for the indentation) and a methodology where the change swelling of the beads upon dissociation of the carboxyl groups was used to estimate the elastic response of the cellulose network in the beads. In the present work, this was complemented by a macroscopic elasticity measurement of the dried and reswollen beads using a specially developed methodology, described in detail in the experimental section (in SI). These measurements are all representing different responses of the beads when they are dried together, and they are all important for the development of the molecular contact between the cellulose surfaces. In Table SI.1, in Supporting Information, the results from all of these measurements are summarized. The trend for all measurements is the same, i.e., as expected, the modulus is decreased with the charge and, hence, water-induced swelling of the beads.

The AFM measurements, in Table SI.1, represent the elastic properties of the outermost surface of the beads, while the macroscopic modulus describes the response of the entire bead when compressed. Finally, the swelling response modulus shows how the cellulose network inside the beads responds when the charges of the oxidized beads are dissociated. Previous investigations^20^ have demonstrated that the external and internal portions of cellulose fibers have a modulus of 0.01–1 and 0.1–5 MPa, respectively, which are in fair agreement with the values in Table SI.1, indicating that the wet beads can serve as excellent models for the drying and consolidation of cellulose-joints.

Surface modification and coatings can significantly alter the adhesion between cellulose joints. In this respect, the LbL assembly is a common method used to controllably modify cellulose surfaces through the sequential addition of oppositely charged polymer or nanoparticle layers.^21−25^ Here the LbL addition of poly(allylamine hydrochloride) (PAH) and hyaluronic acid (HA) onto model cellulose thin films was monitored by stagnation point adsorption reflectometry (SPAR)^26^ to determine how much polyelectrolyte that is adsorbed to the surfaces. This is essential to allow for a fair interpretation of the collected adhesion results

Figure 2a shows how the adsorption, measured as Δ*S*/*S*_0_, increases with the addition of each polyelectrolyte. The adsorbed mass (mg/m^2^), as presented in Figure 2b, was determined by a multilayer optical model.^26^Figure 2a,b clearly demonstrates that increasing the charge density of the cellulose increases PE adsorption.^27^ In addition, the thickness of the dry thin films was measured with AFM (Figure 2c). These measurements show a nonlinear growth of the film thickness following PE layer adsorption which correlates well with previously reported results.^21^ Increased adsorption with the charge density was further observed via AFM.

**Figure 2:**
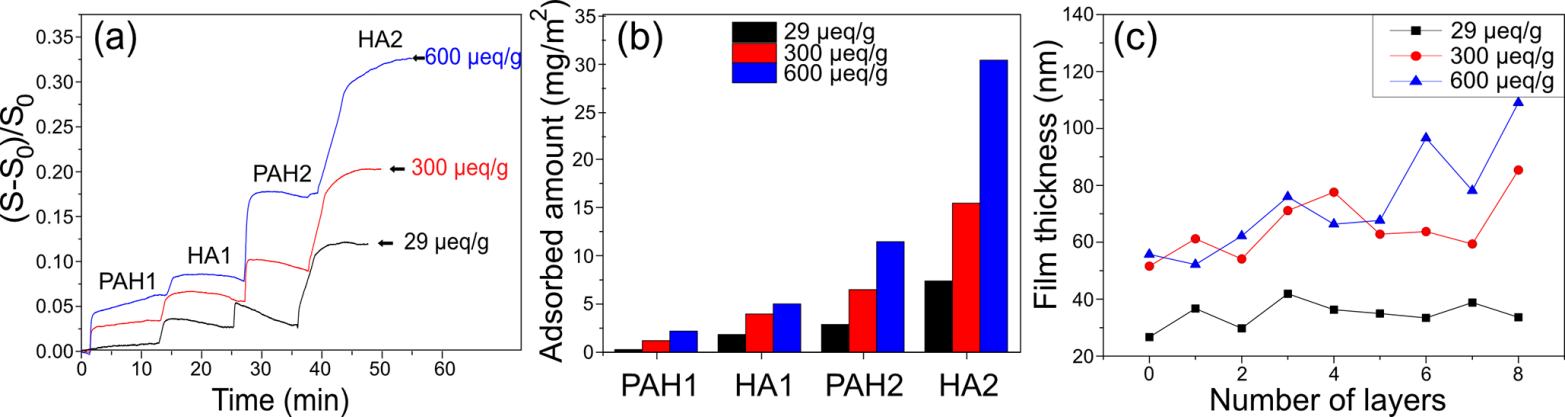
Data from the reflectometry measurements showing the adsorption of 2 bilayers of PAH and HA (a), the determined adsorbed amount for each single layer (b), and the thickness of the cellulose with the adsorbed film measured with AFM (c).

The macroscopic mechanical properties of the LbL-treated beads are shown in Figure SI.1 in the Supporting Information. The elastic modulus of the wet beads is not significantly affected by LbL polyelectrolyte adsorption. This is to be expected, as the adsorbed polyelectrolyte layers contribute to less than 0.01% of the total thickness of the cellulose bead (100 nm thick adsorbed layer following 8 bilayers surrounding a 1000 μm reswollen bead).

While LbL adsorption has limited impact on the macroscopic mechanical properties, it is however expected that the adsorbed PEs will change the surface structure of the beads. The surface morphologies of the dry, highly charged cellulose beads with and without surface treatment were imaged via AFM, and the results are summarized in Figure 3. Following polyelectrolyte adsorption, the roughness of the beads significantly increases, with small nanoscale globular structures of unmodified beads being replaced by large (>1 μm) assemblies. The roughness measurements show a continuous increase in roughness from 5 nm for the untreated bead (0 bilayers) up to 183 nm for beads treated with 10 bilayers. Importantly, this is not necessarily the surface structure of the wet beads since the difference in elastic properties between the cellulose and the adsorbed PE layers is well-known to induce buckling or wrinkling of the outermost layers during drying.^28^ However, as was demonstrated earlier,^21,29^ it is also likely that the adsorbed PE layers also will have a wet structure, in addition to this drying-induced structure, that will significantly affect the adhesion between surfaces.

**Figure 3:**
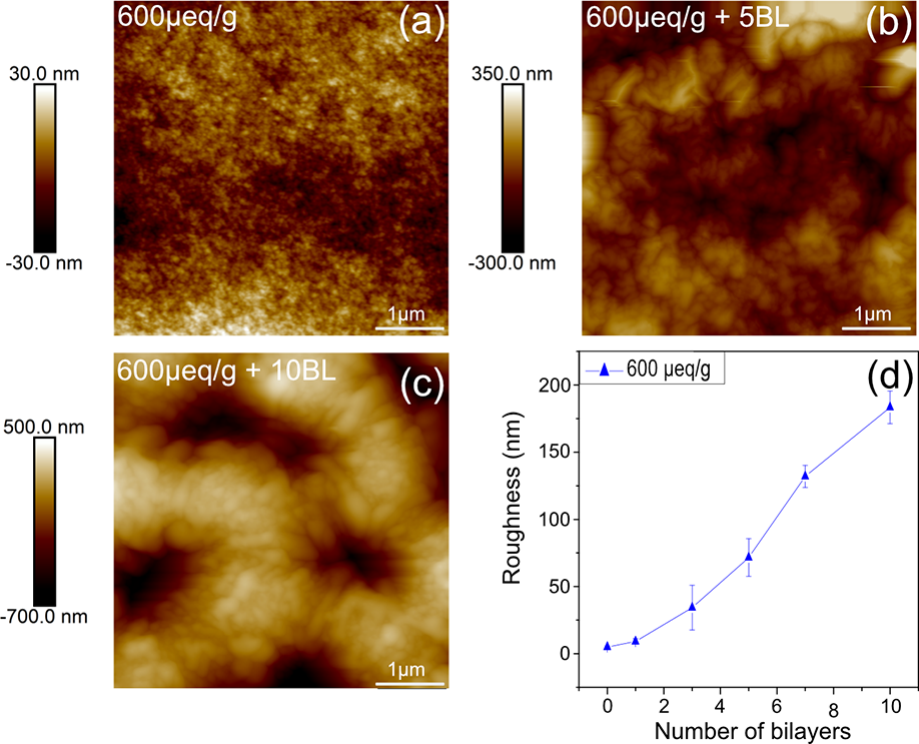
AFM height images of the surface of the dry high charge cellulose beads before and after LbL treatment (a–c) and dry surface roughness measurements (d) as a function of the added number of bilayers of PAH and HA.

The structure of the treated surfaces, as observed with AFM (Figure 3), is further supported by SEM images shown in Figure 4, where the LbL-treated surfaces show wrinkling and a much rougher surface (Figure 4d–f) compared with the untreated beads (Figure 4a–c). The spherical geometry of the cellulose beads is advantageous since it allows for the visualization of the contact area before separation, and the development of the contact zone can be monitored during the drying. For the untreated cellulose beads (with a cellulose charge of 600 μeq/g), the contact area appears smooth and sealed (Figure 5c).

**Figure 4:**
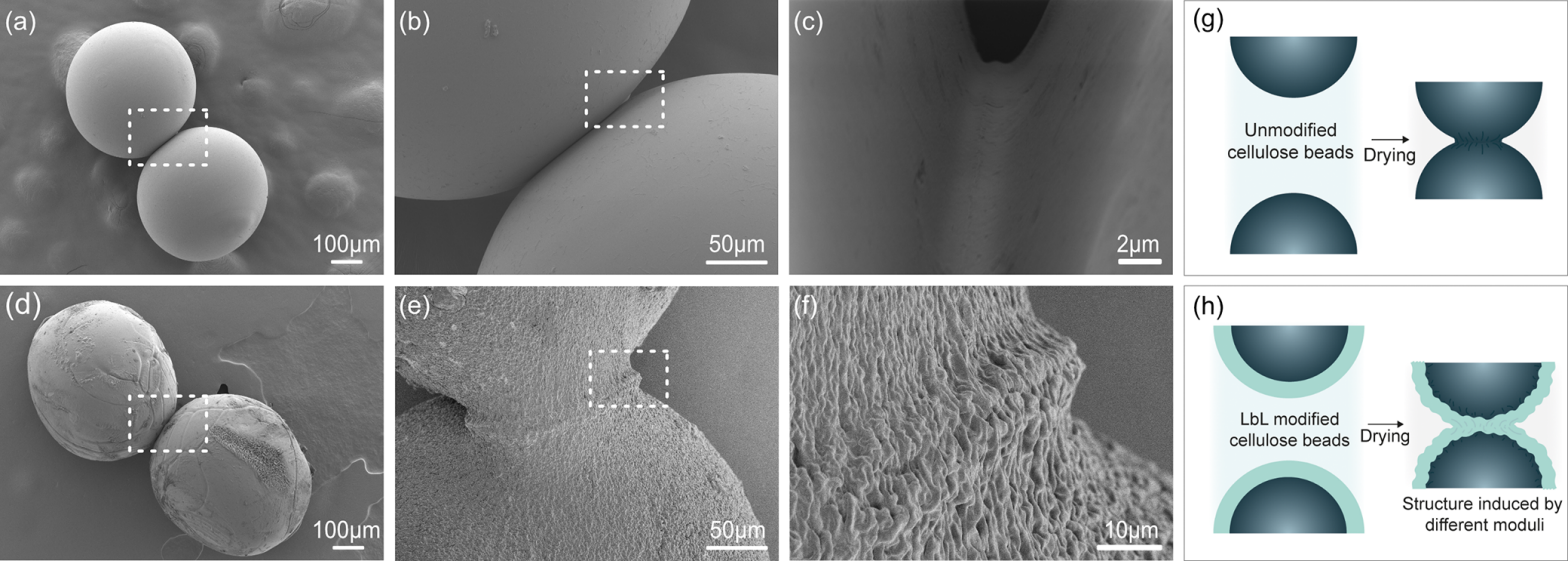
SEM images of the dry bead joints with and without LbL treatment. (a) Shows the untreated low charge density bead joints, while (b) and (c) show closeups of the joint area for these beads. (d) Shows the LbL-treated high charge density beads with 5 bilayers of PAH and HA, while (e) and (f) show closeups of the joint area for these treated beads. (g, h) Schematic descriptions of the structure change of the unmodified beads and LbL-coated beads. (g) Shows the case of untreated cellulose beads, and (h) shows the structure of the interphase between the LbL-treated cellulose beads also inducing a change in the outer layer of the cellulose.

**Figure 5:**
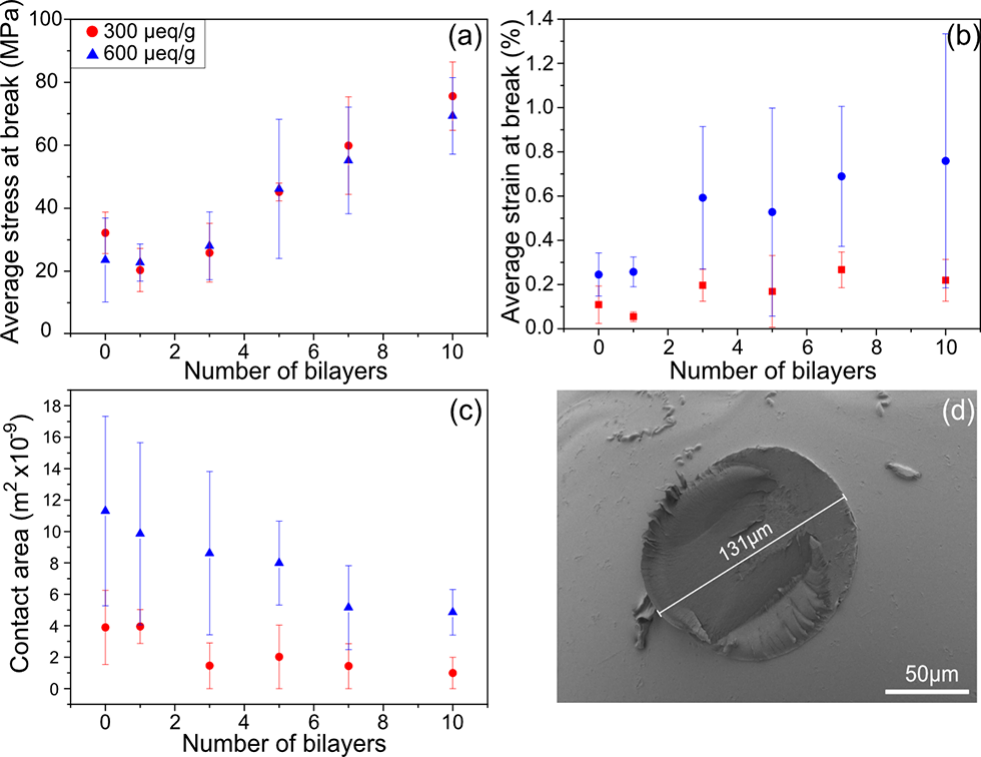
Stress and strain at break of the bead joints as a function of the added number of bilayers of PAH and HA (a, b) and the corresponding contact area measurements after separation of the beads (c). (d) SEM image of the contact area after the separation of two untreated beads.

Comparatively, the LbL-treated beads (Figure 4d–f) are significantly rougher and appear to have a stretched or turgid contact zone. The structure of the contact zone can be attributed both to a structure of the PE layers themselves as well as to the incompatible, with respect to the wet modulus of the materials, interfacial drying of the PE layers, and the supporting cellulose bead. At higher resolutions, more intriguing details of the contact zone can be observed (Figure 4f). It is obvious that, apart from the structure found on areas that are not in contact, the drying of the two LbL-containing beads has initiated a larger deformation of the contact zone with wrinkles at 90° to the contact area. It also appears that the macroscopic contact zone is smaller than the contact zone for the untreated surfaces.

Before fully evaluating the adhesive properties of these joints, it is hence necessary to describe, in more detail, what is happening upon drying two LbL-treated surfaces together. According to the AFM and SEM results, the LbL treatment, 5 bilayers, results in an around 70 nm thick LbL structure on top of the cellulose surface. As this sandwich structure is drying, the slightly rougher LbL film will shrink and wrinkle. Consequently, the structure of the cellulose surface is altered. This is schematically illustrated in Figure 4h. This means that the cellulose bead and, naturally also, cellulose fibers treated in the same way will have built-in stresses due to the inhomogeneous shrinking of the two materials, i.e., LbL-film and cellulose. When these LbL structures are formed on the beads, as illustrated in Figure 4h, the drying and formation of the joint induces a shrinkage of the contact zone that is now significantly rougher than the contact between the nontreated surfaces (Figure 4g). It is also obvious that the pull-off force between these surfaces will be dependent on (a) the adhesion between the cellulose and the LbL film, (b) the built-in stresses due to the uneven shrinkages of the two materials, (c) the mechanical properties of the cellulose and the LbL film, and (d) the extension of the structure formed at the interface. In turn, this also means that direct contact between the cellulose surface is very limited in the case of “thick” LbL films. This necessary insight into the details of the contact between the modified cellulose surfaces would be impossible without these model studies, and more measurements are needed to elucidate the optimum composition of additives and the needed level of modification of the cellulose surface.

In order to evaluate the stress at break when separating the beads, the contact zones were evaluated after separating the surfaces together with the force at separation, and these results are shown in Figure 5. The average stress at break was similar for both medium and highly charged beads and increased with an increasing number of adsorbed polyelectrolyte layers (Figure 5a). In addition, the strain at break showed an overall increasing trend, especially for the high-charge joints (Figure 5b). Moreover, the contact area decreased with an increasing number of polyelectrolyte layers, with the medium-charged beads showing the overall lowest contact area (Figure 5c). This might be related to the fact that high-charge beads are softer (Figure SI:1) than medium-charge beads and thus are more conformable and able to form a larger contact area.

Low-charged (29 μeq/g) beads were significantly stiffer than medium- and high-charged beads (Figure SI.1) and thus could not form an effective joint when dried together.

To further investigate the effect of the surface treatment on the formation of the contact area between the cellulose-rich surfaces, the LbL-treated cellulose bead joints were placed onto carbon tape to dry and introduce strain between the beads (Figure 6a,b). During conventional drying on a Teflon surface, the cellulose beads shrink and move closer together to form a closely packed contact area (Figure 4). However, when the beads are attached to the tape and thus restrained from moving, the strong interactions within the contact area prevented a movement of the beads leading to necking and stretching of the polymers in the polymer complexes in the LbL film (Figure 6b,c). At high resolution the contact area shows a very thin threadlike structure being pulled at the interface of the polymer-rich surfaces (Figure 6d). These findings support and actually explain the results from previous studies,^12^ where the adhesive properties of the same LbL system were investigated and illustrated but could not be fully explained. The extremely long-range interaction in the AFM colloidal probe measurements^21^ for surfaces containing these types of LbLs were left unexplained, but the current results demonstrate that long-range interactions are due to the stretching of the polymer complexes formed at the interface in the LbL film. This also again shows the details that can be identified with these new model materials.

**Figure 6:**
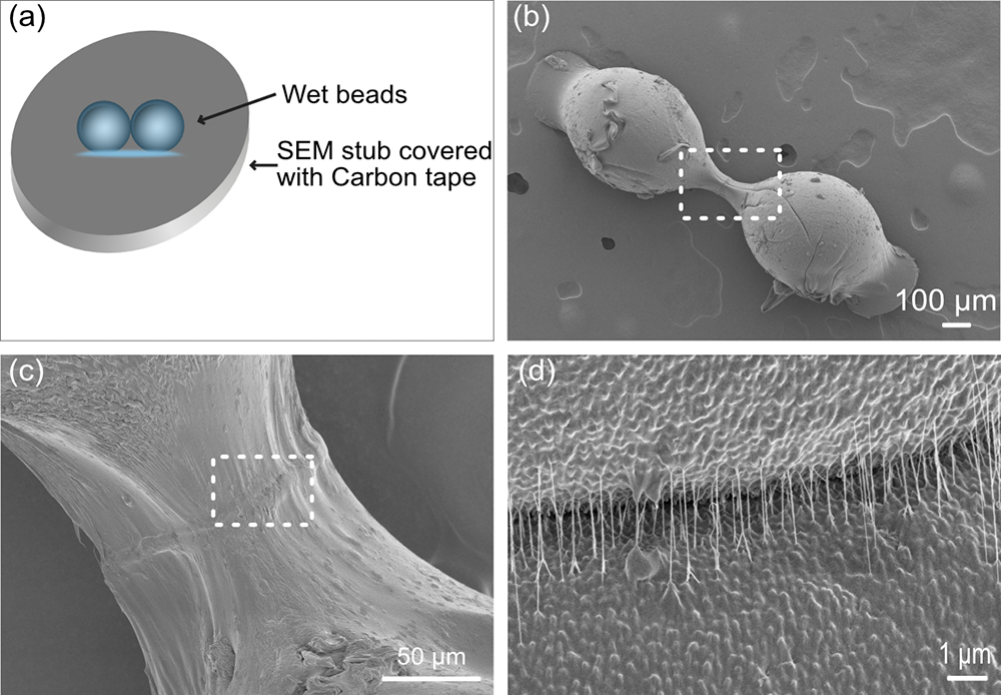
(a) Schematic description of the drying of the beads. (b–d) SEM images of the LbL-treated bead joints dried on carbon tape (b) and closeups of the joint area in (c) and (d) showing the formation of necking of the contact zone (b, c) between the beads and the strings of polymer complexes created at the interfaces (d).

Microscopy and adhesion measurements show the fascinating effect that strength additives have on cellulose joints and clearly demonstrate that there is a complicated interplay between the properties of the supporting beads, the structure of the added layers, and the changes that occur during drying. All of these factors contribute to the size and properties of the formed contact zone (modulus, stress, strain at break, etc.), which all can be probed using the model cellulose joints presented here.

All this, hence, shows that the regenerated cellulose beads can be used as excellent model materials to investigate the mechanisms by which cellulose-rich surfaces form the initial contact and finally dry together to a joint and how surface modification impacts the formation and strength of the contact area. Specifically, by using smooth cellulose spheres, the surface structure and interface of the joints could be visualized before and after separation. By sequentially adsorbing PAH and HA via a LbL assembly, a rough and structured surface was created, allowing for a more intimate contact at the interface. These added layers influence the properties of the cellulose beads as well as the formation of the contact zone between the surfaces during drying. Additionally, upon separation, the polymer chain disentanglement in the wet/moist state leads to a high separation force and a large separation distance of the LbL-treated cellulose surfaces due to the stretching of the polymer complexes formed at the interface. These detailed insights would not have been possible to elucidate without the technique presented in this work.

## Supporting Information Available

Experimental section, mechanical properties of the beads, and AFM images of the cellulose films and the bead’s surface (PDF)
