# Supplementary material for: The Use of Model Cellulose Materials for Studying Molecular Interactions at Cellulose Interfaces
Source: ACS Macro Lett. 2023 Nov 1;12(11):1530–5. doi: 10.1021/acsmacrolett.3c00578 (PMC10666532; doi:10.1021/acsmacrolett.3c00578)
Supplement: Supplementary file 1 — mz3c00578_si_001.pdf [file mz3c00578_si_001.pdf]

SUPPORTING INFORMATION

# The Use of Model Cellulose Materials for Studying Molecular Interactions at Cellulose Interfaces

Nadia Asta,* Michael S. Reid, Torbjörn Pettersson, and Lars Wågberg*

*nadiaa@kth.se

*wagberg@kth.se

## Experimental

**Materials**

Never-dried dissolving grade, fully delignified and bleached, softwood pulp fibres containing 95.4% cellulose were provided by Domsjö Fabriker AB. The pulp fibres were washed before use, with deionized water to remove metal ions and dissolved colloidal substances. Dimethylacetamide (DMAc), Lithium Chloride (LiCl), Hydrochloric acid (HCl), N-methylmorpholine-N-oxide (NMMO, 50 wt% in $H_2O$) and Dimethyl sulfoxide (DMSO) were all purchased from Sigma Aldrich Sweden AB. Sodium chloride (NaCl), Polyallylamine hydrochloride (PAH) (Molecular weight of 17 kDa) and Hyaluronic acid (Molecular weight of 1.5-1.8 x$10^6$ Da) were, also, purchased from Sigma Aldrich Sweden AB and used as received.

**Model materials**

1. **Regenerated cellulose beads**

Cellulose beads were prepared from a Cellulose/DMAc/LiCl solution and a carboxymethylated Cellulose/DMAc/LiCl solution (to introduce a higher charge), according to a method developed previously[1–9]. To start off, carboxymethylation of the dissolving pulp fibres was performed following the same exact protocol which was explained in detail in a previous publication[4]. The charge density of the unmodified dissolving pulp was 29µeq/g and the obtained charge densities after carboxymethylation are 300µeq/g and 600 µeq/g. After that, both unmodified dissolving pulp and carboxymethylated pulp fibres were subjected to the same dissolution protocol. 1.5g of washed pulp (either carboxymethylated or non-modified pulp) were swollen in water for 1 hour and then the fibres were filtered and submerged in ethanol which was exchanged twice a day for two days. Next, the ethanol was exchanged with DMAc. The fibres were allowed to swell in DMAc which, similarly, was replaced twice a day for two days. On the $5^{th}$ day, 98ml DMAc was dehydrated by heating it in an oil bath at 110°C for an hour. At the same time, 7g of LiCl were dried in an oven at 105°C. After one hour, the temperature was turned off and the LiCl was mixed with the DMAc and was allowed to dissolve completely while the temperature decreases. When the temperature of the mixture reached 40°C, the filtered fibres were added to the DMAc/LiCl mixture and were left to stir overnight at 4°C. A completely transparent 1.5wt% Cellulose/DMAc/LiCl solution was formed. The dissolved solution was later filtered using a 45 µm acrodisc PTFE-filter (VWR, Sweden) and then the precipitation was performed using a syringe pump dripping the solution at a rate of 40µl/min into a bath of Ethanol. The gel beads were formed and left to stabilize in ethanol overnight then the ethanol was exchanged with milli-Q water in order to wash the gel beads and completely remove the solvent. The washing step was performed at least 3 times a day for 7 days. The beads were stored in milli-Q water at 21.4°C and 17.9% RH. In a later step, the beads were dried on a Teflon film for several hours at 21.4°C and then reswollen in milli-Q water before use in the next experiments.

2. **Regenerated cellulose films**

The regenerated cellulose thin films were prepared as follows and used as a model material for SPAR and AFM measurements. Dissolving pulp fibres were dissolved in NMMO/DMSO mixture according to a previously described method[10–12]. The Cellulose/NMMO/DMSO solution was reheated to 125°C and spin-coated onto oxidized and PVAm-treated silica wafers. The cellulose thin films were then regenerated in milli-Q water, washed and heat treated at 105°C for a couple of hours to ensure aqueous stability. These cellulose films were stored in Petri dishes at 21.4°C until use. The thickness of the thin films was measured in a later step using AFM and the procedure is explained in the methods section below.

**Methods**

1. **Stagnation point adsorption reflectometry (SPAR)**

The adsorbed amount of Polyallylamine Hydrochloride and Hyaluronic Acid were measured using Stagnation point adsorption reflectometry (SPAR; Laboratory of Physical Chemistry and Colloidal Science, Wageningen University).
For this, regenerated cellulose thin films were prepared as described above and used as a model material[11]. The adsorption using SPAR equipment is explained in detail by Dijt at al[13]. In short, the change in refractive index at the stagnation point upon adsorption of polyelectrolytes is measured and the obtained signal is then converted into an adsorbed amount which was calculated using an optical model software (Huygens software; Dullware, The Netherlands) as included in the software to the SPAR equipment. For this, the dn/dc values of the respective components were measured using refractometry and the thickness of the cellulose thin films before and after adsorption of polymer layers were measured via AFM by performing a scratch test as described below. SPAR experiments were conducted at 24°C and 31% RH. The pH was fixed at 8 and the flow rate at 1 mL/min. The polyelectrolytes were used at a concentration of 0.1g/L and 10mM Sodium chloride solution was used as solvent.

**2. Surface modification of the cellulose beads**

The cellulose gel beads were modified by adsorbing layers of PAH and HA on the surface by applying the Layer-by-Layer modification technique. To carry out this procedure, a syringe was utilized to contain the cellulose gel beads, and a porous aluminium foil covered the fluid path to secure the beads without obstructing liquid flow. A Teflon tube was connected to the syringe's needle hub, and a clamp was employed to regulate the addition and removal of the polyelectrolyte (PE) solutions. Both PE solutions (PAH and HA) were prepared at a concentration of 0.1g/L, with the addition of 10mM NaCl.
The procedure consisted of the following steps. The cellulose gel beads were initially placed inside the syringe and submerged in a 10mM NaCl solution. The NaCl solution was then replaced with PAH solution to facilitate the adsorption of the first cationic layer onto the beads. After adsorption, the PE solution was removed, and the beads were rinsed with a 10mM NaCl solution for a few minutes. Subsequently, the second anionic layer was adsorbed by substituting the solvent with HA solution, followed by another rinsing step. The adsorption time for each PE layer was 5 minutes. At this stage, one bilayer had been successfully adsorbed onto the surface of the beads. The above process was repeated multiple times until the desired number of bilayers was achieve.

**3. Elasticity measurements of the cellulose beads**

The elasticity of the gel beads was measured using a modified version of the Contact adhesion Tester[3,14]. More specifically, the gel bead was fully submerged in a 10mM NaCl solution in a flat petri dish which was placed on an analytical balance. The latter was used to record the applied load while compressing the bead with a flat metal rod (10mm diameter) which is connected to a moving linear stage at a rate or 10μm/s. When moving this rod in the liquid a capillary force and a buoyancy force will give additional response to the analytical balance and this was subtracted from the measured load. The corrected force as a function of the displacement was fitted in the force range of 5 to 25 mN to a linearized Hertz method (Carl and Schillers 2008; Hellwig et al. 2017) according to:

$$F = \frac{1}{2}\left(\frac{4}{3}\frac{E}{1-\nu^2}\right)\left(\frac{D}{2}\right)^{1/2}\left(\frac{h}{2}\right)^{3/2} \quad (1)$$

$$F^{2/3} = \frac{1}{2}\left(\frac{4}{3}\frac{E}{1-\nu^2}\right)^{2/3}\left(\frac{D}{2}\right)^{1/3} h \quad (2)$$

$$F^{2/3} = slope * h \quad (3)$$

$$slope = \frac{1}{2}\left(\frac{4}{3}\frac{E}{1-\nu^2}\right)^{2/3}\left(\frac{D}{2}\right)^{1/3} \quad (4)$$

$$E = \left(\frac{2*slope}{\left(D/2\right)^{1/3}}\right)^{3/2} \frac{3}{4}(1-\nu^2) \quad (5)$$

In the above equations (1) to (5), *F* is the corrected force detected by the analytical balance, *E* is the Young's modulus, *D* is the diameter of the wet bead, ν is the Poisson ratio (a value of 0.3 was used), *h* is the indentation, e.g. related to the motion of the linear stage, corrected with an approximate point of contact. Eq (1) is the Hertz model for compression of a sphere between two flat surfaces. Eq (2) is a linearization of Eq (1). The force in the power of (2/3) in the indentation regime of the measurement is linear fitted versus *h* to obtain

the *slope* in Eq (3). From Eq (1) – (3) the slope of such fitting is related to *E*, *D* and ν according to Eq (4) The value of this *slope* is therefore used to calculate the Young's modulus according to Eq (5). The beads were compressed up to a maximum load of 10g which corresponds to about 20-30% compression of the full size of the bead. At least 3 samples were tested for each type of bead. Besides giving the elasticity, this experiment provided also a precise measurement of the wet diameter of the cellulose beads which is reported in Figure SI.1 (b) below.

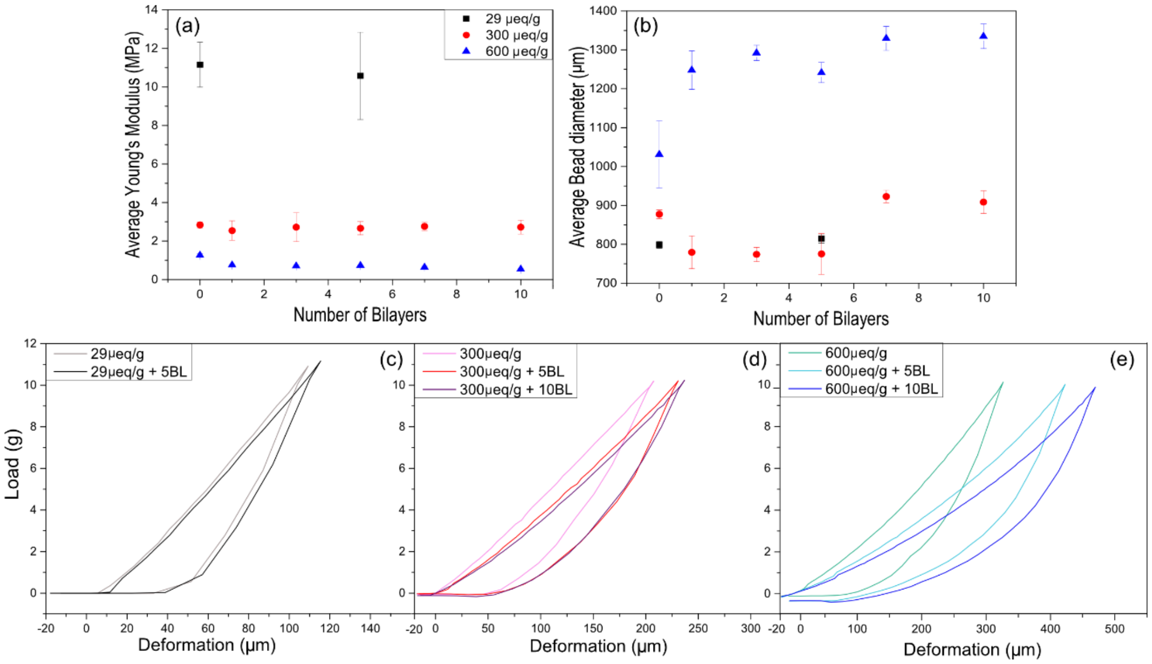


Figure SI.1: Elasticity data showing the measured Young's Modulus of the gel beads with different charge densities and increasing number of adsorbed bilayers of PAH and HA (a) and the corresponding bead diameters in (b). Figures (c-e) display the load vs deformation curves of some representative samples of each charge density before and after LbL treatments, zero in deformation is arbitrary set prior to the increase in load due to the compression, the absolute zero in deformation is not crucial for the calculation of the Young's modulus, due to the use of the linearized Hertz model.

**Table SI.1. Evaluation of different elastic properties of the wet cellulose beads. [a]These data are reproduced from reference[15].**

| Charge of the cellulose (μeq./g) | 29 (No oxidation) | 300 | 600 |
|---|---|---|---|
| AFM indentation (kPa)a | 45 | 38 | 30 |
| Macroscopic modulus (MPa) | 10 ±0.516 | 3 ±0.327 | 1 ±0.003 |
| Swelling response (kPa)a | - | 110 | 70 |

## 4. Scanning electron microscopy

The dry size of the cellulose beads was measured using a low vacuum Tabletop Scanning Electron Microscopy (Hitachi TM-1000, Japan).

Field-Emission Scanning Electron Microscopy FE-SEM (Hitachi S-4800, Japan) was used to characterize the surface morphology of the beads as well as the formed model joints. Prior to imaging the cellulose beads and filaments, the samples were coated with a thin layer of platinum/palladium using a Cressington 208 HR high-resolution sputter coater.

## 5. Atomic Force Microscopy

An AFM (Bruker Multimode 8) was used to measure the thickness of the cellulose thin films before and after adsorption of a few polyelectrolyte layers. For this experiment, NTESP (Bruker, Camarillo, CA) cantilever with a resonance frequency of 300kHz and a force constant of 40N/m was used in contact mode in air. The silica wafer containing the dry cellulose film was scratched with a sharp blade. Then, 10x10μm scans were performed covering a part of the film and the scratched area on 4 different spots on the surface, as shown in Figure SI.2. This measurement was initially performed for the untreated cellulose films with three different charge densities

(29µeq/g, 300µeq/g and 600µeq/g). After that, eight layers of PAH and HA were adsorbed (10 minutes adsorption time for each layer) and the 4 scans were repeated after each layer of adsorbed polymer to measure the thickness of the added LbLs. The films were blow dried with compressed air before each measurement. The results of the thickness measurements can be seen in Figure 3.

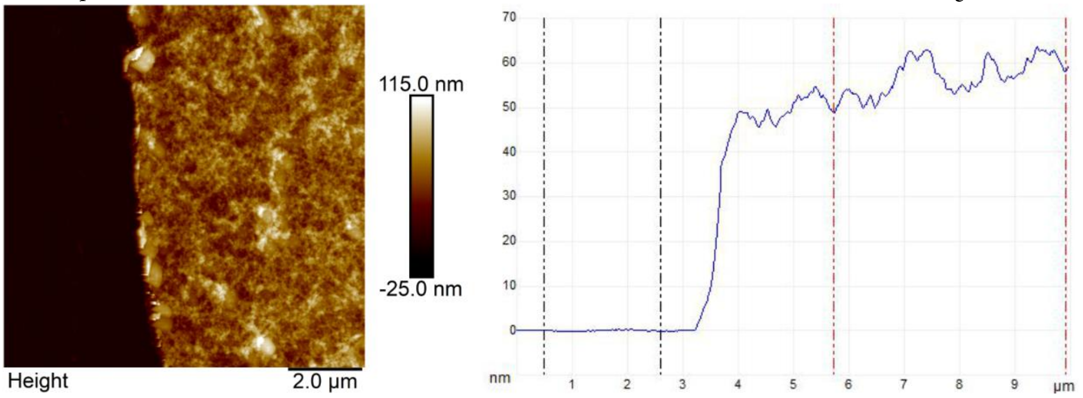


Figure SI.2: AFM Height scan on a scratched cellulose thin film on the left and an illustration of how the thickness of the film is analyzed with the AFM Nanoscope analysis software.

AFM was also used to characterize the surface roughness of the dry cellulose beads before and after Layer-by-Layer treatment. For this, RTSP-150 cantilever with a resonance frequency of 150kHz and a force constant of 6N/m, was used in tapping mode. The cellulose gel beads were dried on a Teflon film prior to the measurement. Next, they were placed on an AFM holder covered with double-sided tape to hold the dry beads in place while measuring surface topography to be used for the surface roughness calculation. This measurement was performed mainly on the high charged cellulose beads since they were considered the most interesting samples to further investigate, the images from the high charged beads are presented in Figure 4, while an image of low charge cellulose bead is shown in Figure SI.3.

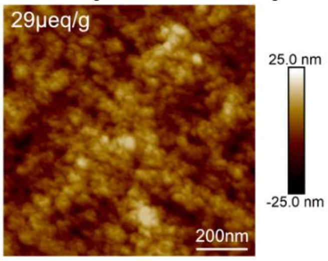


Figure SI.3: AFM height image of the low charge untreated cellulose bead.

**6. Model joints formation**

The regenerated cellulose gel beads were placed on a Teflon film to dry for several hours. Then, they were reswollen in milli-Q water for at least 1 day. Next, a pair of cellulose beads were placed to dry again, laying on top of each other, on a PDMS coved glass slide, as shown in Figure1 in the main text. The model joint is created upon drying of the cellulose gel beads at 21.4°C and 17.9% RH. Since cellulose adheres to PDMS[15], the latter is used to stabilize the gel beads during the drying process which supports the formation of the model joint[17]. The cellulose beads were left to dry for several hours and then, the formed bead joints were stored in a petri dish to be studied in a later step.

**7. Mechanical testing of the bead joints**

The dry cellulose bead joint was carefully placed onto the pull-off stage, as illustrated in Figure SI.4 below, using a micromanipulator. Then, the upper and lower beads were glued to glass slides, which were fixed onto the pull-off stage, using UV curing glue (Norland Optical Adhesive 81). A UV light pen (Inova X5) was used to cure the UV glue for at least 60 seconds and the process was monitored using a digital microscope (AM7013MZT, Dino-Lite Premier Digital Microscope). Next, the motor of the pull-off stage is moved upwards, at a rate of 1um/sec, while recording the load and the position of the motor until separation of the two beads. The contact area after separation of the bead joint was imaged with an optical microscope and later measured using ImageJ software.

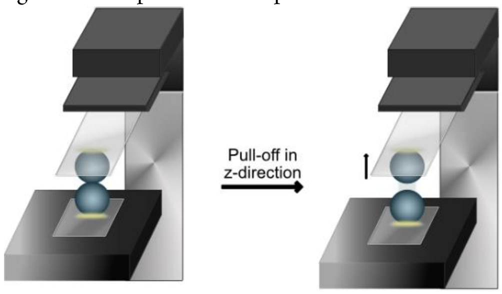

Figure SI.4: Illustration of the tensile test performed on the bead joints.

## References


(1) Carrick, C.; Pendergraph, S. A. Nanometer Smooth, Macroscopic Spherical Cellulose Probes for Contact Adhesion Measurements. *ACS Appl Mater Interfaces* **2014**. https://doi.org/10.1021/am505673u.

(2) Li, H.; Mystek, K.; Wågberg, L.; Pettersson, T. Development of Mechanical Properties of Regenerated Cellulose Beads during Drying as Investigated by Atomic Force Microscopy. *Soft Matter* **2020**, *16* (28), 6457–6462. https://doi.org/10.1039/d0sm00866d.

(3) Li, H.; Roth, S. V; Freychet, G.; Zhernenkov, M.; Asta, N.; Wågberg, L.; Pettersson, T. Structure Development of the Interphase between Drying Cellulose Materials Revealed by In Situ Grazing-Incidence Small-Angle X-Ray Scattering. *Biomacromolecules* **2021**, *22* (10), 4274–4283. https://doi.org/10.1021/acs.biomac.1c00845.

(4) Karlsson, R. M. P.; Larsson, P. T.; Yu, S.; Pendergraph, S. A.; Pettersson, T.; Hellwig, J.; Wågberg, L. Carbohydrate Gel Beads as Model Probes for Quantifying Non-Ionic and Ionic Contributions behind the Swelling of Delignified Plant Fibers. *J Colloid Interface Sci* **2018**, *519*, 119–129. https://doi.org/10.1016/j.jcis.2018.02.052.

(5) Köklükaya, O.; Karlsson, R. M. P.; Carosio, F.; Wågberg, L. The Use of Model Cellulose Gel Beads to Clarify Flame-Retardant Characteristics of Layer-by-Layer Nanocoatings. *Carbohydr Polym* **2021**, *255*. https://doi.org/10.1016/j.carbpol.2020.117468.

(6) Li, H.; Kruteva, M.; Mystek, K.; Dulle, M.; Ji, W.; Pettersson, T.; Wågberg, L. Macro- And Microstructural Evolution during Drying of Regenerated Cellulose Beads. *ACS Nano* **2020**, *14* (6), 6774–6784. https://doi.org/10.1021/acsnano.0c00171.

(7) Träger, A.; Klein, G.; Carrick, C.; Pettersson, T.; Johansson, M.; Wågberg, L.; Pendergraph, S. A.; Carlmark, A. Macroscopic Cellulose Probes for the Measurement of Polymer Grafted Surfaces. *Cellulose* **2019**, *26* (3), 1467–1477. https://doi.org/10.1007/s10570-018-2196-2.

(8) Mystek, K.; Reid, M. S.; Larsson, P. A.; Wågberg, L. In Situ Modification of Regenerated Cellulose Beads: Creating All-Cellulose Composites. *Ind Eng Chem Res* **2020**, *59* (7), 2968–2976. https://doi.org/10.1021/acs.iecr.9b06273.

(9) Mystek, K.; Li, H.; Pettersson, T.; Françon, H.; Svagan, A. J.; Larsson, P. A.; Wågberg, L. Wet-Expandable Capsules Made from Partially Modified Cellulose. *Green Chemistry* **2020**, *22* (14), 4581–4592. https://doi.org/10.1039/d0gc01523g.

(10) Gunnars, S.; Wågberg, L.; Cohen Stuart, M. A. Model Films of Cellulose: I. Method Development and Initial Results. *Cellulose* **2002**, *9* (3–4), 239–249. https://doi.org/10.1023/A:1021196914398.

(11) Aulin, C.; Ahok, S.; Josefsson, P.; Nishino, T.; Hirose, Y.; Österberg, M.; Wågberg, L. Nanoscale Cellulose Films with Different Crystallinities and Mesostructures - Their Surface Properties and Interaction with Water. *Langmuir* **2009**, *25* (13), 7675–7685. https://doi.org/10.1021/la900323n.

(12) Benselfelt, T.; Cranston, E. D.; Ondaral, S.; Johansson, E.; Brumer, H.; Rutland, M. W.; Wågberg, L. Adsorption of Xyloglucan onto Cellulose Surfaces of Different Morphologies: An Entropy-Driven Process. *Biomacromolecules* **2016**, *17* (9), 2801–2811. https://doi.org/10.1021/acs.biomac.6b00561.

(13) Dijt, J. C.; Stuart, M. A. C.; Fleer, G. J. Reflectometry as a Tool for Adsorption Studies. *Adv Colloid Interface Sci* **1994**, *50* (C), 79–101. https://doi.org/10.1016/0001-8686(94)80026-X.

(14) Gustafsson, E.; Johansson, E.; Wågberg, L.; Pettersson, T. Direct Adhesive Measurements between Wood Biopolymer Model Surfaces. *Biomacromolecules* **2012**, *13* (10), 3046–3053. https://doi.org/10.1021/bm300762e.

(15) Carl, P.; Schillers, H. Elasticity Measurement of Living Cells with an Atomic Force Microscope: Data Acquisition and Processing. *Pflugers Arch* **2008**, *457* (2), 551–559. https://doi.org/10.1007/s00424-008-0524-3.

(16) Hellwig, J.; Karlsson, R. M. P.; Wågberg, L.; Pettersson, T. Measuring Elasticity of Wet Cellulose Beads with an AFM Colloidal Probe Using a Linearized DMT Model. *Analytical Methods* **2017**, *9* (27), 4019–4022. https://doi.org/10.1039/c7ay01219e.

(17) Rundlöf, M.; Karlsson, M.; Wågberg, L.; Poptoshev, E.; Rutland, M.; Claesson, P. Application of the JKR Method to the Measurement of Adhesion to Langmuir-Blodgett Cellulose Surfaces. *J Colloid Interface Sci* **2000**, *230* (2), 441–447. https://doi.org/10.1006/jcis.2000.7108.
